# Delivery Room Quality Improvement Project Improved Compliance with Best Practices for a Community NICU

**DOI:** 10.1038/srep37397

**Published:** 2016-11-21

**Authors:** Charles W. Sauer, Mallory A. Boutin, Aayah N. Fatayerji, James A. Proudfoot, Nabil I. Fatayerji, David J. Golembeski

**Affiliations:** 1 Department of Pediatrics, University of California, San Diego, San Diego, CA, United States; 2 Osteopathic Medicine, Western University of Health Sciences, Pomona, California, United States; 3 Clinical and Translational Research Institute, University of California, San Diego, San Diego, CA, United States

## Abstract

A Quality Improvement bundle was implemented with the goal of standardizing the multidisciplinary approach to delivery room management. We used a Pre-Post Quality Improvement initiative with the following aims: (1) Placement of a functioning pulse oximeter by two minutes after birth, (2) Delayed intubation, (3) Normothermia on Neonatal Intensive Care Unit Admission, (4) Use of a pre-brief, debrief, and delivery room checklist. Data was collected for 548 infants, which represents every admission to the Palomar Rady Children’s Hospital Neonatal Intensive Care Unit during the 35 month study period from January 1, 2010 to November 30, 2012. The intervention began on May 1, 2011. The objective of increasing the frequency of each goal was met. A significant decrease in rates of retinopathy of prematurity in our post-intervention group was found. Odds ratio 0.00 (0.000, 0.696) p = 0.008. However, this was not confirmed in the multivariable analysis so should be interpreted with caution. This quality improvement project had a positive effect on newborn resuscitation at Palomar Medical Center.

The transition from fetus to neonate is a time of significant physiologic adaptation, especially for babies born prematurely^1^,^2^. This period of transition has been referred to as the “Neonatal Golden Hour” and begins in the delivery room. Interventions during this transition from fetus to neonate have been shown to have a great influence on an infant’s future morbidities^2^,^3^,^4^. Therefore, optimizing delivery room care for newborns can improve both short term and long term outcomes. The goal of this quality improvement (QI) project is the implementation of a standardized multidisciplinary approach to delivery room management.

## Methods

**Figure 1:**
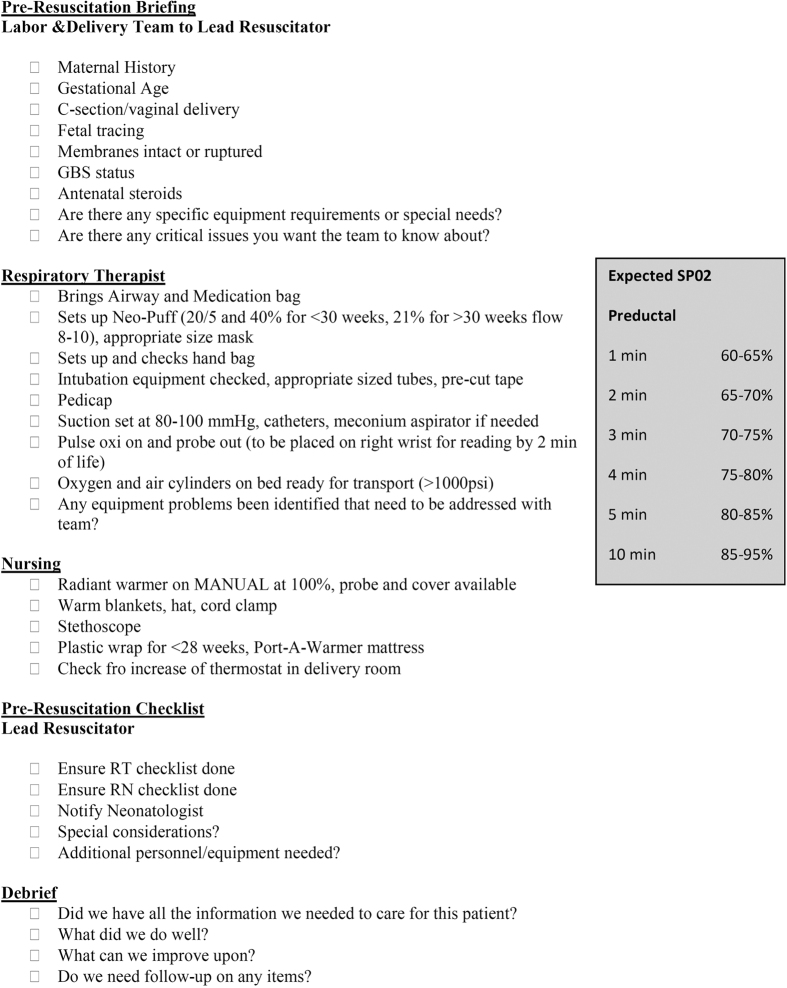
Delivery room checklist.

An expert panel identified the need for delivery room resuscitation improvements in a network of neonatal intensive care units. Several evidence-based best practice interventions along with corresponding implementation goals to improve neonatal outcomes before, during, and after delivery room resuscitation were chosen. Based on the recommendation of the panel, training was done for the delivery room team in order to achieve the following goals: (1) Placement of a functioning pulse oximeter by two minutes after birth to facilitate supplemental oxygen administration, (2) Delayed intubation in preference of using continuous positive airway pressure (CPAP) with the goal of surfactant avoidance in the delivery room, (3) Normothermia at Neonatal Intensive Care Unit (NICU) admission (temperature between 36.5 and 37.5 degrees celcius), (4) Promotion of teamwork and communication between the obstetrician, labor and delivery staff, and the neonatal resuscitation team via the use of a team pre-brief, debrief, and delivery room checklist (Fig. 1). Pre intervention data was retrospectively collected for infants born January 1, 2010 up to the initiation of the intervention. The intervention began on May 1, 2011 and ended November 30, 2012. All infants born at Palomar Medical Center for whom the high risk delivery team was called during the study period and who were admitted to the Palomar Rady Children’s Hospital NICU participated in this collaborative study through the California Perinatal Quality Care Collaborative (CPQCC).

Data was extracted from patient medical records. The statistical program “R” was used to analyze the data.

This study was performed in accordance with relevant guidelines and regulations. The Institutional Review Board at Rady Children’s Hospital approved this project and the Institutional Review Board at Palomar Medical Center exempted a full review and both institutional review boards waived informed consent since this was a quality improvement project.

## Results

Data was collected for 548 infants. Of which, 249 were born prior to the start of the intervention and 299 were born after the start of the intervention. This represents all the infants for whom the high risk delivery team attended the delivery and then were subsequently admitted to the Palomar Rady Children’s Hospital NICU during the 35 month study period. Demographic data for the infants is shown in Table 1.

Compliance with O_2_ saturation monitoring by two minutes after birth improved during the intervention period. At baseline, approximately 26% of infants overall had O_2_ saturation monitoring by two minutes after birth. This improved to almost 55% (p = 0.007) by the close of the study period. Prior to the intervention there was not documentation of when or if a pulse oximeter was placed, and therefore we could not compare this intervention with the pre-intervention group to the post-intervention group.

The percentage of infants who were intubated in the delivery room significantly decreased from 14.1% prior to the intervention to 5.4% after the intervention. (p < 0.001). As the intervention period progressed, the odds of intubation in the delivery room decreased by an average of 4.6% per month but this was not found to be statistically significant (p = 0.322).

The percentage of infants who were given surfactant in the delivery room decreased from 2.8% prior to the intervention to 1.0% after the intervention, but this was not found to be a significant decrease (p = 0.198). There was also a non-significant trend downward of infants who received surfactant in the delivery room as the intervention period progressed. (p = 0.789).

The percentage of infants who had a normal temperature on NICU admission significantly increased from 78.3% prior to the intervention to 86.3% after the intervention. (p < 0.017). As the intervention period progressed the rates of normothermia on NICU admission increased from 83% at baseline to 89% at the end of the study period. Although rates were trending in a positive direction for this measure, these results are not statistically significant (p = 0.36).

An upward monthly trend during the intervention period was noted for compliance with the use of the checklist and a team pre-brief and debrief from 25% at baseline to 92% at the end of the intervention (p < 0.001). Prior to the intervention there was not documentation of whether or not a checklist was used or if a team pre-brief or debrief took place, and therefore we could not compare this intervention with the pre-intervention group to the post-intervention group.

Outcome data was collected for occurrence of respiratory distress syndrome, bronchopulmonary dysplasia, death, patent ductus arteriosus, pneumothorax, necrotizing entercolitis, retinopathy of prematurity, post-hemorrhagic hydrocephalus, intraventricular hemorrhage, and length of stay. A univariable and multivariable logistic regression was performed. In the univariable logistic regression a significant decrease in retinopathy of prematurity was found for the post-intervention group. Odds ratio 0.00 (0.000, 0.696) p = 0.008. No other outcomes were found to be statistically significant. A multivariable logistic regression was performed and none of the outcomes were found to be statistically significant. In addition there was not enough data to fit bronchopulmonary dysplasia, death, retinopathy of prematurity, or post-hemorrhagic hydrocephalus into the multivariable logistic regression.

**Figure 2:**
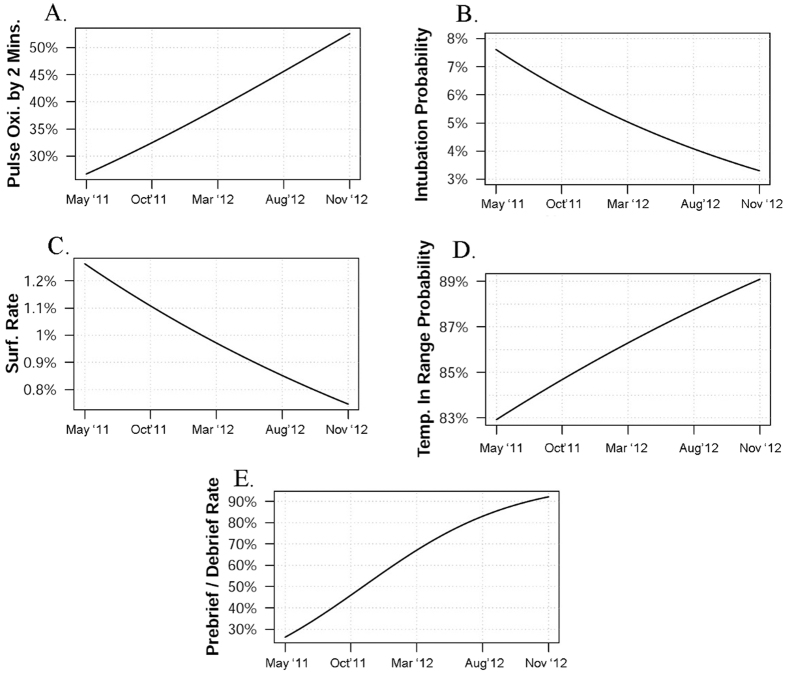
Graphs of the study period showing (**A**). Increasing percentage of infants who had O2 saturation monitoring by 2 minutes of age, (**B**). Decreasing intubation rate, (**C**). Decreasing use of surfactant in the delivery room. (**D**) Increased number of infants with a normal temperature on NICU admission, (**E**) increasing use of a checklist and holding a pre-brief and debrief session.

The data comparing interventions prior to the intervention compared to after the start of the intervention is shown in Table 2. Intervention improvements during the study period are shown in Table 3. Quality improvement goal trends are shown in Table 4. Table 5 shows the results of the univariable logistic regression for outcomes, and graphical representation of each intervention is shown in Fig. 2.

## Discussion

By participating in this CPQCC collaborative quality improvement initiative, the primary goal for the Palomar Rady Children’s Hospital NICU was to compare the effectiveness of a bundle of delivery room interventions with previous standard of care techniques in an attempt to improve newborn outcomes at our facility. We chose a comprehensive QI program in the hope that patient outcomes may be more positively impacted than if a single quality measure was selected^5^. In addition, we chose multiple interventions because no single intervention can fully address complex quality concerns; multifaceted approaches are preferred so that many small improvements will result in an additive or multiplicative positive impact for the patient^6^.

Our first approach to quality improvement in delivery room management was the placement of pulse oximeters within the first two minutes after birth. Early monitoring of oxygen saturation for infants in the delivery room is key to establishing appropriate oxygenation levels and preventing morbidities associated with both over and under oxygenation, especially for preterm infants^7^. Pulse oximetry is a particularly important type of monitoring because it provides an objective and continuous measure of both oxygenation and heart rate, the two key indicators of a neonate’s need for interventions^8^.

Despite several large studies in recent years, the optimal range for oxygen saturation in preterm infants remains unclear^9^,^10^. Hyperoxia often results in retinopathy of prematurity and bronchopulmonary dysplasia, while hypoxia can lead to necrotizing enterocolitis, hearing loss or impairment, negative neurodevelopmental outcomes, and death^7^. In order to minimize morbidity and mortality, the concentration of supplemental oxygen in the delivery room should be closely monitored starting at birth and actively titrated against the patient’s oxygen saturation as measured by pulse oximetry^8^.

We found a significant decrease in rates of retinopathy of prematurity in our post-intervention group in the univariable logistic regression. The use of a pulse oximeter may have limited our oxygen use and this finding is biologically plausible. However, this finding was not confirmed in the multivariable logistic regression and so needs to be interpreted with caution.

Our second QI aim was to decrease the rate of intubation in the delivery room with the aim of delaying the procedure until NICU admission. Preterm infants frequently need respiratory assistance and approximately 10% of all newborns will require some type of ventilatory support, although finding the right level of support can be difficult^11^. Despite the fact that endotracheal intubation and surfactant therapy has been shown to significantly decrease the rates of death, air leak and death or bronchopulmonary dysplasia^12^,^13^, in recent years there has been a push to switch to a strategy of less invasive ventilation and to delay intubation.

There are risks inherently involved with the intubation process. A recent prospective study looked at 273 intubation encounters and found that an adverse event occurred in 39% of the encounters, hypoxemia occurred 44% of the time, and bradycardia occurred 24% of the time^14^. Another recent retrospective chart looked at 88 extremely low birth weight infants that were intubated in the delivery room during the first ten minutes after birth. Those infants who needed more than one attempt at intubation for successful placement of the endotracheal tube had a greater likelihood of death or neurodevelopmental impairment^15^. A randomized control trial from 2006 showed that successful intubations with fewer attempts occur more often in infants who received premedication which included a paralytic agent^16^. A consensus statement from the International Evidence-Based Group for Neonatal Pain concluded that “tracheal intubation without the use of analgesia or sedation should be performed only for resuscitation in the delivery room or for life-threatening situations associated with the unavailability of intravenous access”^17^. Therefore there should be some benefit to delaying intubation until the baby gets back to the NICU where intravenous access can be established and premedications given.

In addition, there may be some benefit to avoiding intubation altogether. The COIN trial, SUPPORT, and a Vermont Oxford Network trial showed that CPAP was a viable and safe alternative to intubation and surfactant^18^,^19^,^20^. A retrospective analysis found an association between the number of intubation attempts and intraventricular hemorrhage in preterm infants^21^. In a recent meta-analysis, mechanical ventilation was shown to be a risk factor for bronchopulmonary dysplasia, a significant morbidity in the preterm population^22^. Therefore, strategies of preventing or delaying intubation, may have some benefit. We were successful in decreasing our intubation rate and surfactant rate in the delivery room during the study period.

The third aim of this project was to increase the occurrence of normothermia at NICU admission. The neonatal resuscitation program guidelines recommend infant temperature to be maintained between 36.5 and 37.5 degrees celcius^11^. Newborn infants commonly experience a drop in body temperature immediately post-birth, a phenomenon that is particularly evident in preterm infants^3^. Preterm infants struggle with maintaining body temperature due to heat loss associated with high surface area to volume ratio, underdeveloped epidermis, a limited subcutaneous fat layer, and poor vasomotor control^23^. Neonatal hypothermia is associated with various poor outcomes including development of respiratory distress syndrome, necrotizing enterocolitis, intraventricular hemorrhage, and death^3^,^24^. Several strategies for maintaining appropriate body temperature have been suggested including occlusive wrapping, immediate use of hats, heated mattresses, radiant warmers, and kangaroo care^25^. For this project, we employed a combination of these strategies. Maintaining normothermia improves outcomes and can be easily addressed through the use of targeted quality improvement initiatives^26^.

Our final aim was to increase teamwork and cooperation among the various patient care teams in the delivery room via pre-briefs, debriefs, and checklists. Checklists have been used for years in the aviation industry to reduce errors and improve passenger safety^27^. In the healthcare setting, checklists during high risk deliveries have been shown to reduce communication errors and help clinicians rapidly identify problems^28^. In a survey of 15 hospitals, clinicians perceived checklists as beneficial especially for the preparation of equipment^29^.

During a neonatal resuscitation is it important that all of the participants present work well together as a team. Simpson *et al*. found that team communication is an important factor in leading to effective teamwork^30^. One of the benefits of a pre-brief is that it allows team members to initiate their communication early in the process and facilitates the assignment of team roles.

The United States military has used after-action review or debriefs for decades in order to improve learning and performance^31^. A meta-analysis of debriefs found that organizations can improve individual and team performance by approximately 20 to 25% by using properly conducted debriefs^32^. We were able to improve our use of prebriefings, debriefings and checklists during the study period.

There are some limitations associated with this study. This was a relatively small study with only 548 infants. Also, about 45% of the infants in the study were term which made it more difficult to find a change in many of our outcomes which have more of an impact on infants of lower gestational age. There is bias in the assessment of whether the team pre-brief and debrief were used because it was reported by the resuscitation leader. In addition we need to consider the inherent limitations of interpreting the results of a study that was not done in a blinded randomized controlled fashion.

We met our delivery room quality improvement goals of increasing the percentage of infants who had O2 saturation monitoring by two minutes of age, decreasing our intubation rate, decreasing our use of surfactant in the delivery room, increasing the use of a checklist and holding a pre-brief and debrief session. A significant decrease in rates of retinopathy of prematurity in the post-intervention group was found, however this was not confirmed in the multivariable analysis so should be interpreted with caution. Overall, joining this quality improvement collaborative had a positive effect on newborn resuscitation at Palomar Medical Center. As we move forward, there is a need to continue to identify best practice interventions for the delivery room so that they can be incorporated into everyday use.

## Additional Information

**How to cite this article**: Sauer, C. W. *et al*. Delivery Room Quality Improvement Project Improved Compliance with Best Practices for a Community NICU. *Sci. Rep.* **6**, 37397; doi: 10.1038/srep37397 (2016).

**Publisher’s note:** Springer Nature remains neutral with regard to jurisdictional claims in published maps and institutional affiliations.

## Figures and Tables

**Table 1:** Patient Demographics.

	Pre-Intervention	Post-Intervention
n	249	299
Male n, (%)	134, (54%)	157, (53%)
Gestational Age in weeks mean,(SD), median, (IQR), range, % of population <37 weeks	35 6/7, (3.73), 35 5/7, (33.86, 39), 23 2/7–42 3/7, 56.6%	36, (3.37), 36, (34, 39), 24–42, 55.5%
C-section n, (%)	144, (58%)	185, (62%)
1 minute Apgar Score, median, (IQR)	8, (6, 8)	8, (6, 8)
5 Minute Apgar Score, Median, (IQR)	9, (8, 9)	9, (8, 9)
Birth Weight, Mean, (SD)	2630.9, (911.3)	2685.3, (916.9)

**Table 2:** Occurrence of Delivery Room Interventions Before and After Initiation of the Quality Improvement Project.

	Mean before (n)	Mean after (n)	Odds Ratio	95% Confidence Interval	P value
Intubation Rate	14.1% (35)	5.4% (16)	0.345	(0.173, 0.659)	<0.001*
Surfactant Use in the Delivery Room	2.8% (7)	1.0% (3)	0.350	(0.058, 1.551)	0.198
Normal Temperature on Admission	78.3% (195)	86.3% (258)	1.741	(1.089, 2.799)	0.017*
Functioning Pulse Oximeter by 2 Minutes after Birth	Data not available**	38%(114)	—	—	—

^*^P < 0.05.

^**^The use of a pulse oximeter in the delivery room was not documented until after the start of the intervention.

**Table 3:** Intervention Improvements during the Study Period.

	Estimate	Std. error	Odds Ratio	95% Confidence Interval	P value
Functioning Pulse Oximeter by 2 Minutes	0.056	0.021	1.057	(1.016, 1.101)	0.007*
Intubation Rate	−0.044	0.045	0.957	(0.877, 1.044)	0.322
Surfactant use in the Delivery Room	−0.027	0.099	0.974	(0.082, 1.183)	0.789
Normal Temperature on Admission	0.026	0.028	1.026	(0.971, 1.085)	0.360
Prebrief/Debrief and Checklist Completed	0.175	0.025	1.191	(1.135, 1.250)	<0.001*

Note: A logistic regression model was used with date as a continuous covariate.

^*^P < 0.05.

**Table 4:** Trend Direction of Intervention.

Intervention	Trend	Goal Met?
Functioning Pulse Oximeter by 2 Minutes	Increased	Yes
Intubation Rate	Decreased	Yes
Surfactant use in the Delivery Room	Decreased	Yes
Normal Temperature on Admission	Increased	Yes
Prebrief/Debrief and Checklist Completed	Increased	Yes

**Table 5:** Univariable Logistic Regression for Patient Outcomes.

	Mean before (n)	Mean after (n)	Odds Ratio	95% Confidence Interval	P- value
Respiratory Distress Syndrome	67.1% (167)	72.6% (217)	1.299	(0.885, 1.906)	0.190
Bronchopulmonary Dysplasia	1.2% (3)	0.0% (0)	0.000	(0.000, 2.001)	0.093
Death	0.8% (2)	0.0% (0)	0.000	(0.000, 4.430)	0.206
Patent Ductus Arteriosus	3.2% (8)	1.7% (5)	0.511	(0.130, 1.798)	0.269
Pneumothorax	4.4% (11)	2.7% (8)	0.593	(0.203, 1.648)	0.345
Necrotizing Enterocolitis	1.2% (3)	0.7% (2)	0.551	(0.046, 4.846)	0.663
Retinopathy of Prematurity	2.4% (6)	0.0% (0)	0.000	(0.000, 0.696)	0.008*
Post-hemorrhagic hydrocephalus	0.8% (2)	0.0% (0)	0.000	(0.000, 4.430)	0.206
Intraventricular Hemorrhage	3.6% (9)	1.3% (4)	0.362	(0.080, 1.317)	0.096
Length of Stay	17.8 days	16.8 days	—	—	0.934

Note: Significance is determined by Fisher’s exact test for each binary variable and the Mann-Whitney U test for length of stay.
